# Holding a foster child’s mind in mind: study protocol for a cluster-randomized controlled trial of mentalization-based therapy (MBT) for foster families

**DOI:** 10.1186/s40359-023-01103-8

**Published:** 2023-03-07

**Authors:** Nina Thorup Dalgaard, Anne Marie Anker Villumsen, Kresta Munkholt Sørensen, Nick Midgley, Mette Skovgaard Væver, Mathilde Almlund, Maiken Pontoppidan

**Affiliations:** 1 VIVE- The Danish Center for Social Science Research, Herluf Trolles Gade 11, 1052 Copenhagen, Denmark; 2 Department of Social Work, Faculty of Social Education, Social Work and Administration, University College Copenhagen, Kronprinsesse Sofies Vej 35, 2000 Frederiksberg, Denmark; 3 Anna Freud Centre/University College London (UCL), 4-8 Rodney St, London, N1 9JH UK; 4 Department of Psychology, University of Copenhagen, Østerfarimagsgade 2A, 1350 Copenhagen K, Denmark

**Keywords:** Foster care, Mentalization-based family therapy, Attachment, Effectiveness

## Abstract

**Background:**

Children in foster care are psychologically vulnerable and show more social, developmental, and behavioral problems than those living with their family of origin. Many foster parents struggle to care for these children, some of whom have experienced severe adversity. Research and theory suggest that developing a strong and supportive foster parent–child relationship is essential for foster children to become more well-adjusted and experience a decrease in behavioral problems and emotional maladjustment. Mentalization-based therapy (MBT) for foster families aims at increasing the reflective functioning of the foster parents, thus promoting the development of more secure and less disorganized child attachment representations, which is subsequently proposed as a factor that reduces behavioral problems and emotional maladjustment in children and promotes their overall well-being.

**Methods:**

This is a prospective cluster-randomized controlled trial with two conditions: (1) the intervention group participating in MBT, and (2) the control group who receive usual care. Participants are 175 foster families with at least one foster child aged 4–17 years with emotional or behavioral problems. The intervention will be offered to foster families by 46 foster care consultants from 10 municipalities in Denmark. The foster care consultants will be randomized to MBT training (n = 23) or usual care (n = 23). The primary outcome is the psychosocial adjustment of the foster child measured by the Child Behavior Checklist (CBCL) as reported by foster parents. Secondary outcomes include child well-being, parental stress, parent mental health, parent reflective function and mind-mindedness, parent/child relations, child attachment representations, and placement breakdown. In order to explore implementation fidelity as well as practitioner experiences, we will administer questionnaires designed for this study and conduct qualitative research exploring the practice of the MBT therapists.

**Discussion:**

This trial is the first experimental study of a family therapeutic intervention based on attachment theory for foster families within the Scandinavian context. This project will contribute with novel knowledge on attachment representations in foster children and the effects of an attachment-based intervention on essential outcomes for foster families and children.

*Trial registration* ClinicalTrials.gov NCT05196724. Registered on January 19, 2022.

## Background

Children in foster care are psychologically vulnerable and show more social, developmental, and behavioral problems than children who live with their family of origin [1, 2]. Many foster children have experienced adverse events before their initial placement, such as physical and emotional abuse and neglect [3]. Furthermore, studies show that children in out-of-home care face significantly higher risks of mental health problems [4, 5], drug and alcohol abuse [6], and suicidal behavior [4, 7, 8] when compared to the general population. Removing a child from their family of origin and placing them in foster care can be a traumatic event for the child and, in some cases, does not improve a child’s life-course [9]. Research is equivocal about the long-term effects of foster care, with studies finding both negative and positive effects on foster children’s developmental trajectories, as measured by behavioral, emotional, and mental health outcomes [9–11].

While a secure, long-term placement is associated with better long-term outcomes for children in foster care [12, 13], placement breakdown is relatively common. Placement breakdown can have devastating consequences for vulnerable children and is costly for society [9, 14]. In Denmark, around 8% of children in foster care experience placement breakdown [15], which typically occurs when foster parents feel unable to care for the child [16]. Many foster parents struggle to manage the complex care needs of the children placed in their care [17]. Therefore, understanding and supporting the needs of foster families is essential if we are to improve the long-term outcomes of children in foster care.

### Attachment representations of children placed in foster care

Theoretically, the difficulties experienced by foster families and the behavioral and emotional symptoms exhibited by the children may be understood through an attachment lens [18]. According to attachment theory, early caregiving experiences influence adaptation and maladaptation across the lifespan by organizing the child’s individual and relational developmental processes “from the cradle to the grave” [19]. Through interactions with the primary caregiver and based on the caregiver’s responses to the child, internal working models of attachment are established early in life. A child will experience grief, anger, and distress as a result of loss of access to existing attachment figures, and this can only be resolved if the child can develop new attachment relationships with alternative caregivers [20]. The child’s attachment pattern can be categorized as either secure or insecure [21], and a secure attachment relationship with a primary caregiver has long-term benefits for children [22]. Some children who experience early trauma, such as harsh neglect and abuse or the loss of their biological parents, may develop disorganized attachment representations, which leaves the children without stable internal working models of intimate relationships. Children who experience abuse and neglect are also at an increased risk of developing reactive attachment disorder (RAD) or disinhibited social engagement disorder (DSED) [23]. Although clinically distinct from each other, the two disorders share common features, and both represent disorders of attachment rooted in early traumatic experiences, which are common among children placed in foster care [23].

Research into child attachment representations and attachment classification of children in foster care is surprisingly scarce. In a meta-analysis of international research, Vasileva and Petermann [24] only identified five studies reporting on child attachment style for children in foster care (n = 255). Only one study was from Europe (the Netherlands), whereas the remaining four were from the US. Based on the meta-analysis, it was estimated that disorganized attachment occurs in 22% of children in foster care and that 43% of children in foster care have an insecure attachment style [24].

Hillman, Cross, and Anderson [25] measured attachment representations by using a story stem approach in which attachment narratives are coded in a dimensional manner. The study compared data from a sample of children placed in foster care with a matched sample of non-maltreated children who lived with their biological parents. The study concludes that the children in the foster care sample consistently displayed more disorganized, avoidant, and negative representations and had significantly fewer representations characteristic of secure attachment [25].

Despite the insights provided by the above studies, there remains a scarcity of research into the attachment representations of children placed in foster care; in a Scandinavian context, until the contribution of the present study, there had been no studies of the attachment representations of children placed in foster care.

### Theory of change in attachment-based interventions

Disorganized attachment representations are highly predictive of later socioemotional maladjustment and behavior problems in children, and similarly, more secure attachment representations are a protective factor for long-term child outcomes [26]. Attachment is a relational and dynamic phenomenon. When a traumatized child is placed in the care of sensitive caregivers who can consistently empathize with the child and understand that the child’s destructive or negative behavior may result from the child's negative prior experiences of abuse and neglect, the child may develop more secure and less disorganized attachment representations [22]. Development of more secure attachment and less disorganized child attachment representations are proposed as factors that can reduce behavioral problems and socioemotional maladjustment symptoms in the children. Understanding and promoting the mechanisms by which foster parents can help children placed in their care to develop more secure and less disorganized attachments is, therefore, an urgent priority [18].

Based on the original conception by Mary Ainsworth, parental sensitivity is defined as a parent’s ability to (1) notice child signals, (2) interpret these signals correctly, and (3) respond to these signals promptly and appropriately [27, 28]. The quality of the attachment bond between child and parent is highly dependent on the sensitivity of the caregiver [22, 29–31]. A growing evidence base indicates that increased parental reflective functioning (PRF) combined with parental sensitivity lead to positive child outcomes [32–34]. Parental reflective functioning refers to the parental ability to hold the child’s mind in mind and understand the child’s behavior as a result of inner thoughts and feelings that may result from early negative experiences [32, 35]. The central mechanism of change in attachment-based interventions is to support the foster parents in meeting the child's needs [36]. This can be done by helping the foster parents to understand the subtle and overt emotional cues in the child's behavior and to respond to these cues in a sensitive and contingent manner.

Reviews of attachment-based interventions for at-risk families with young children find positive effects on parental sensitivity and infant attachment [37, 38]. Reviews examining the effects of attachment-based interventions offered to foster parents and adoptive parents find similar promising results. A narrative review of attachment interventions for foster and adoptive parents and children aged 0–17 years suggests positive effects but also points to the need for larger and more methodologically sound randomized controlled trials of attachment-based interventions that have proven efficacy in biological parent–child dyads [39]. A meta‐analytic review examining the effects of all types of parenting interventions in foster care and adoption finds strong positive effects on sensitive parenting and positive results on dysfunctional discipline, parenting knowledge and attitudes, parenting stress, and child behavior problems. The authors point out that future studies should focus solely on either foster care or adoption populations [40]. Finally, a recent systematic Campbell review explored the effects of attachment-based parenting interventions for foster and adoptive families. The review included 44 studies reporting 27 different samples (19 randomized trials and eight non-randomized studies) [18]. The meta-analyses show improvement in the overall psychosocial adjustment of the child and improvement of positive parent and child behavior and parenting stress post-intervention. Results also show improvement in observed positive parenting behavior 3–6 months after the interventions. Within the review, a meta-analysis was conducted using attachment outcomes. However, in line with the findings regarding the prevalence of attachment representations and attachment styles in foster children, the meta-analysis using attachment as an outcome was inconclusive due to the very limited number of studies that reported on attachment as an outcome (k = 3) [18].

The existing reviews thus show promising results for attachment-based interventions used with foster families. However, there is a need for high-quality RCTs, especially on interventions that target the underlying mechanisms in promoting more secure and less disorganized attachment representations in foster children.

### The intervention: mentalization-based therapy (MBT) for foster families

Most attachment-based parenting interventions are designed for relatively young children [17]. This is reflected in the fact that the mean age of the children included in a recent review of attachment-based interventions was 5.15 years [18]. In Denmark, however, children who are placed in foster care are 11.3 years on average at their first placement [44], and thus there is a strong need for interventions to support foster families with older children. Compared to other attachment-based interventions, MBT can be used with a much broader age range of children. The MBT approach is relatively new to psychological therapy and grew out of developments in psychodynamic therapy and attachment research [41, 42]. Mentalization-based therapy is a short-term semi-manualized treatment. Programs using MBT have been used on many different populations, including parents and babies, adolescents who self-harm, and adults with borderline personality disorder [43–46]. Studies have demonstrated that when parents or foster parents are more sensitive and better able to 'mentalize' their child, these abilities are associated with a range of improved outcomes for children. When used in the context of foster care, MBT aims to promote the quality of relationships, support effective and sensitive foster care, and support foster parents in helping the child understand and manage emotions better. The focus is on improving the core components of secure attachment, particularly by developing reflective functioning for all professionals working with children in out-of-home care. This reflective functioning is assumed to increase the child's psychosocial adjustment and decrease emotional and behavioral problems [33].

Mentalization-based therapy programs consist of up to 12 weekly sessions with the foster parents and child or children covering three core components: (1) psycho-education about mentalizing, trauma, and attachment for foster parents; (2) support for reflective practice in the professional network; and (3) mentalization-based therapy for the foster family [36]. Mentalization-based therapy is a flexible intervention in which therapists can adjust the content and activities within each session to fit the needs of the foster family [36]. Within MBT sessions, the therapists may work with the foster child and parents together or separately. Mentalization-based therapy sessions consist of either therapeutic conversations or activities in which the therapist uses a playful and genuinely curious approach to encourage mentalizing in the foster parents and the child or children. Similarly, the therapists work to prevent breakdowns in mentalizing, which are often caused when families are under stress, by providing a positive example and actively intervening when non-mentalizing dialogue and interactions occur. Therapeutic activities include games, artwork, and role-plays adjusted to fit the child's age, development, and interests [41].

A randomized feasibility study of MBT for foster families in the UK with 36 families found positive results regarding implementation, training of therapists, and acceptance from foster parents. However, it was not possible to estimate a reliable effect size due to the relatively small sample size [41]. The single outcome measure used showed divergent results. Children in the MBT condition reported a large, significant improvement in internalizing symptoms compared to usual care (UC). In contrast, foster parents reported an insignificant improvement in internalizing symptoms favoring the UC group [41]. The study concludes that while the feasibility of the trial had been established, “A full-scale definitive trial with follow-up at the end of all treatments is needed to determine efficacy” [41]. The present trial will be the first experimental study of an attachment-based family therapeutic intervention for foster families and children within the Scandinavian context.

### Research aim

For foster families with children aged 4–17 years, this study examines the effects of MBT, compared to usual care, on child psychosocial adjustment and well-being, parental stress, mental health, reflective function, parental mind-mindedness, and the foster parent–child relationship. The primary outcome is child psychosocial adjustment as measured by the Child Behavior Checklist (CBCL) reported by foster parents and self-report for children aged ten or above. A secondary aim of the study is to provide knowledge about attachment representations in foster children aged 4–11 and how these may be associated with the child's psychosocial adjustment.

We hypothesize that MBT is superior to UC on measures of child mental health and well-being, parent stress, parent–child relationship, foster parent reflective functioning, parental mind-mindedness, and placement stability. We also hypothesize that a high proportion of young foster children will show signs of disorganized and insecure attachment representations that will be associated with psychosocial maladjustment.

## Methods and design

This is a prospective, parallel, cluster-randomised controlled superiority trial with two conditions: [1] an intervention group who will receive MBT and [2] and a control group who receive UC.

**Fig. 1:**
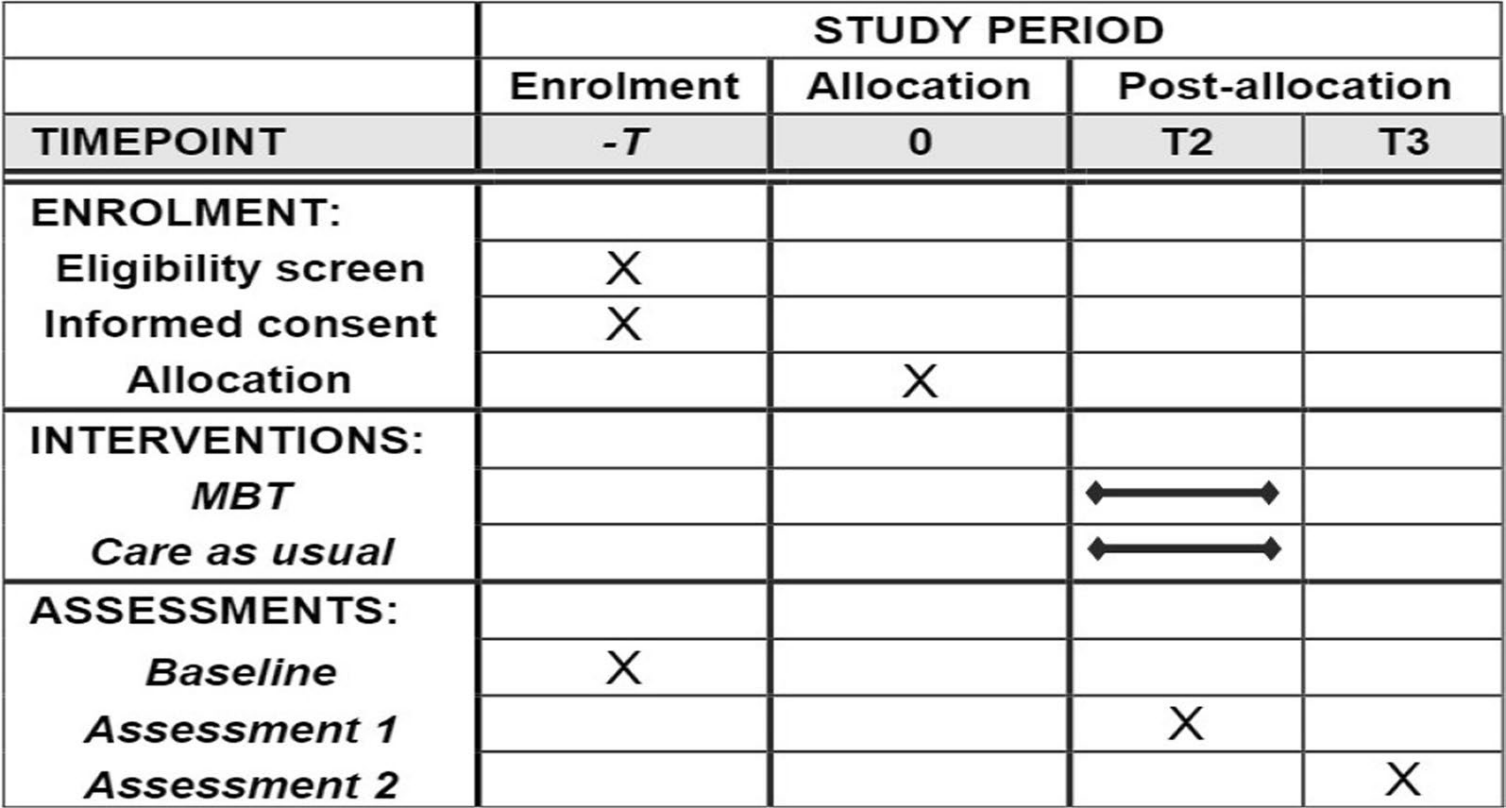
SPIRIT figure

The protocol conforms to the Standard Protocol Items: Recommendations for Interventional Trials (SPIRIT) guidelines (Fig. 1). The final reports of the trial will be written following the Consolidated Standards of Reporting Trials (CONSORT) statement.

### Recruitment and procedures

In Denmark, local foster care placement agencies are part of the municipalities and employ foster care consultants who provide ongoing support for foster parents. Each foster family is assigned to a foster care consultant in their municipality when a child is placed in the family. This assignment is typically based on the availability of time and the current case load of the foster care consultants. Assignments typically take place in a meeting in which the head of the foster care services has the final say on which foster care consultants are assigned to which foster families. If the availability of time or the discretionary assignment to consultants is related to the effect of treatment, this would introduce selection bias in the estimates of treatment effects. However, based on discussions with municipalities, we believe that the assignments to consultants are primarily based on the availability of time, which, according to the municipalities, is not systematically related to consultant quality or treatment group.

**Fig. 2:**
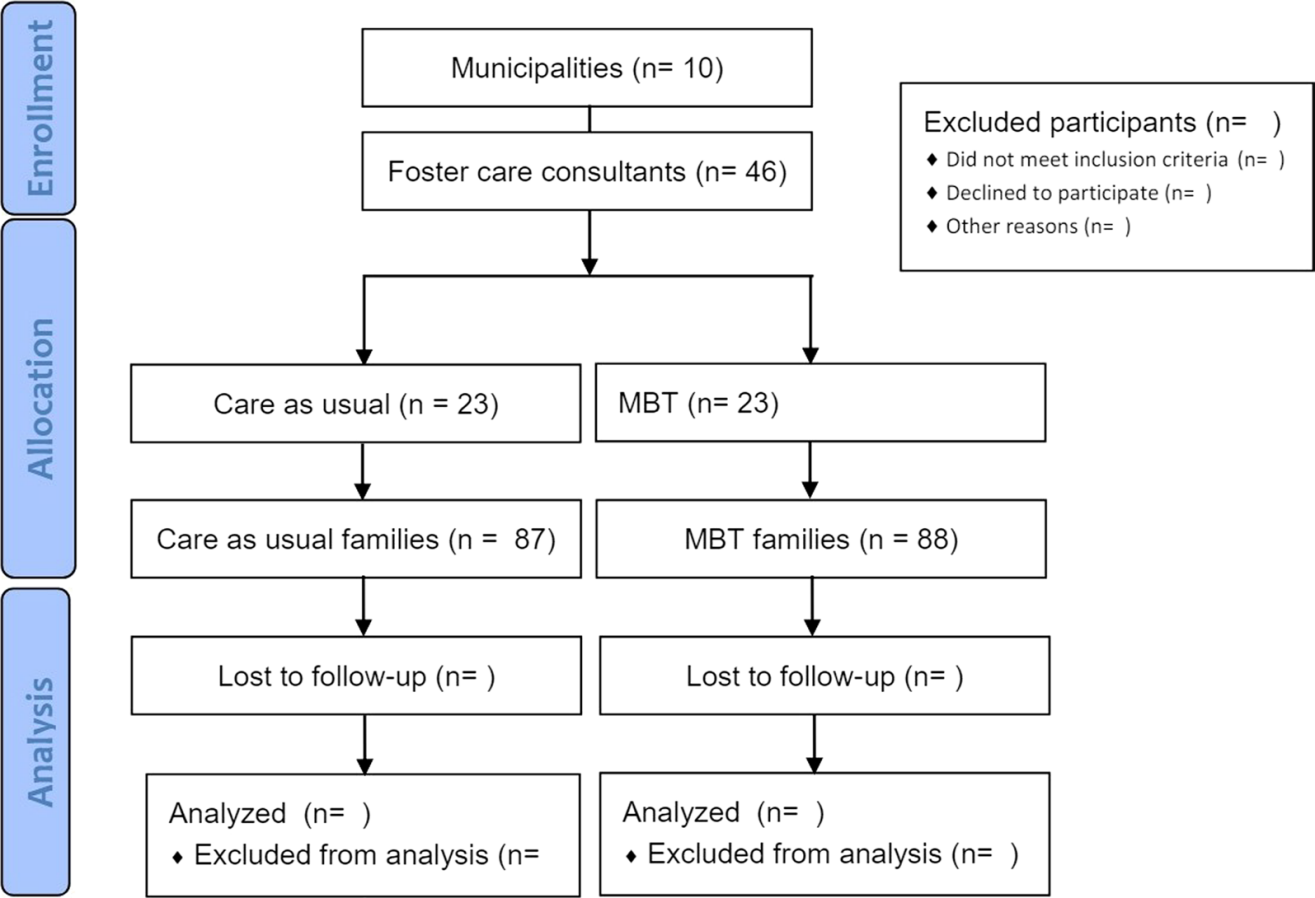
Flow chart

Since each foster care consultant is assigned to a number of foster families we will create a cluster randomized design in which families are randomized to the intervention and control condition based on their assigned foster care consultant witin each municipality. Each participating municipality will be asked to provide an even number of foster care consultants to enter the study. The Danish Center for Social Science Research (VIVE) will receive the names of the foster care consultants and will randomize half of the total number of foster care consultants in each municipality to intervention or control. We will randomize 46 foster care consultants from 10 municipalities across Denmark to MBT training (n = 23) or UC (n = 23). The MBT and control interventions will be offered to 175 foster families by 46 foster care consultants [46 clusters]. The flow chart is presented in Fig. 2.

### Allocation

Due to legal, ethical and practical concerns voiced by the municipalities, it was not possible to apply individual randomization of the foster families. Therefore, we employ a quasi-cluster randomized design in which we randomize foster care consultants into two groups: MBT training or UC. By employing a quasi-cluster randomized design, we minimize the risk of spill-over of the intervention to the control families. Usual care is the service that was offered before the trial. When possible, foster families will receive the MBT intervention or the UC from the foster care consultant they are already familiar with. However, in some rare cases, the local placement agency may need to allocate families to an unfamiliar foster care consultant to ensure an even allocation and comparability between the intervention and control group (e.g., referring families on an alternating basis to treatment and control conditions).

### Recruitment

The participating foster care consultants will screen foster families for eligibility into the trial. The foster families will subsequently be invited to participate in the study by the foster care consultant to whom they were referred. Participation is voluntary. The foster care consultants will give oral information about the trial and give an information leaflet on the study to the foster families. Families can also watch a short video about the study. For legal and ethical reasons, foster families in both conditions will be informed of the condition they are allocated to before consenting to participate in the study. Biological parents who still have custody over children will be asked for consent for the child to participate and foster children aged 15 or above will also be asked for consent. Younger foster children aged 4–14 will be informed of the study in an age-appropriate manner. After receiving consent, the foster care consultant will register the foster parents and children through a registration form.

### Participants

Participants are full-time foster families in Denmark with a foster child aged 4–17. Screening for eligibility will be conducted by municipal foster care consultants and consist of five questions designed for the present study in which the foster parents are asked to indicate if the child has any significant emotional, social, or behavioral problems and if they feel as if they are struggling to care for the child or feel a need for further support in caring for the child. Inclusion criteria are: (1) Any kind of foster family (including professional and kinship care) in one of the participating municipalities with at least one full-time foster child aged 4–17 years; (2) the foster child must have been placed with the foster family for at least four weeks; (3) the target child must display emotional, social, or behavioral problems as reported by the foster parents and the foster parents need to report struggling to care for the child or having a need for extra support in caring for the child. The foster family is excluded if any of the following criteria are present: (1) the foster family is already receiving a family therapeutic intervention; (2) the foster family has previously participated in this study with a different child; or (3) the foster child has formally been diagnosed with autism, psychotic disorder, severe language delay or severe substance abuse problems.

If a family has more than one eligible foster child, both children can participate and receive the intervention with their foster parents. However, for this study, we will only collect data from one target child in each family. The target child will be selected by the foster parents, who will be asked to select the child they are most concerned about.

### Usual care (control group)

As of 2019, foster parents in Denmark receive intensive support during the first six months of a placement consisting of both coursework and supervision from foster care consultants After the first year of placement, training consists of two one-day training courses annually [47]. As part of the follow-up casework, Danish foster parents receive face-to-face counseling from the local authorities at least once every six months [47]. Given their professional and personal skills, counselors can choose whatever counseling methods or techniques they deem best [48]. According to a large survey of services for foster parents and foster children in 50% of Danish municipalities, 35% of foster parents had not received any counseling within the previous year beyond the basic national requirements [49]. Nineteen percent of foster parents had received group supervision, 23% had received supervision by a trained psychologist or psychiatrist, 13% had participated in a peer group for foster parents, and 15% had received other types of supervision or counseling. Many foster children are, at some point during placement, offered an additional intervention besides the placement, such as treatment for mental health issues [49]. One out of three foster parents in the survey [49] reported feeling partially or entirely unequipped to raise children with mental health issues [48, 49]. During the trial, families who receive the MBT intervention will not receive any other therapeutic interventions (including individual psychotherapy for the child). However, they may still receive the regular and mandatory support services available to all foster families in Denmark, consisting of coursework and the twice-yearly supervision. Foster care consultants will record the additional services and interventions received by the foster families in the control group during the trial. Researchers from VIVE will contact the families to motivate them to participate in the data collection.

### Training and provision of the intervention

Training of foster care consultants consists of a one-day online training course followed by a three-day face-to-face interactive course taught by experienced MBT supervisors from the Anna Freud Center. Throughout the trial, two Danish certified MBT therapists and supervisors will deliver monthly group supervision and three booster sessions to the local MBT therapists to ensure fidelity and high quality of the intervention. In order to measure adherence to the intervention, the MBT therapists will complete a short questionnaire designed for the trial to record the content and participants in each session.

Mentalization-based therapy sessions will take place either in the foster families' homes or in a clinical setting, depending on the wishes of the foster parents, and each family will be offered up to 12 sessions over the course of four months.

For the purpose of this trial, we will consider three sessions as the minimal intervention. Families who only received 0–2 sessions will be considered dropouts.

### Treatment fidelity and adherence to the intervention

Therapists in the MBT group will be asked to fill out an adaptation of the MBT adherence scale [50]. In the usual care group, the control foster care consultants will be asked to complete a questionnaire in which they list the additional services offered to the control group families (beyond the mandatory services received by both groups during the same time period as the MBT intervention.

### Data collection

Data will be collected through web surveys at three timepoints: T1: baseline; T2: post-intervention 20 weeks post-baseline, and T3: follow-up 6 months after the end of the intervention. At T1 and T2, we will also request a short video recording and conduct a home visit. For foster families with foster children aged 4–9 years, the data collection will include two home visits at T1 and T2. The home visit will last about an hour. At the home visit, we will conduct an interview (the Story Stem Assessment Profile (SSAP)) with the foster child. The SSAP will be conducted by graduate students in psychology who are thoroughly trained and receive ongoing supervision from a psychologist (first author) with extensive experience of using story stem methods. During home visits, we will strive to ensure that all children understand the purpose of the assessment and consent to participating. All foster families will also be asked to record a six-minute long video where the primary foster parent and the foster child are together. For foster children 8 years and older, the primary foster parent and the foster child will be filmed in a situation where they have been instructed to plan the perfect day together. For foster children aged 4–7 years, the primary foster parent and the foster child will be filmed in a situation in which they have received no special instructions on what to they should do. Foster parents will be asked to choose an activity, that the foster child usually enjoys and to engage in this activity together with the foster child. In foster families with foster children aged 4–9 years old, the video will be recorded during the home visit. Foster families with older children will be asked to record the video sequence and upload it to a secure data depository hosted by the Danish Agency for Governmental IT Services (Statens IT).

Questionnaire data will be collected through secure online survey databases (defgo and SurveyXact). Foster parents will receive an email with a direct link to the questionnaire through Digital Post, a digital mailbox system providing all Danish citizens with a private email account tied to their social security number. Digital Post ensures secure digital communication between Danish citizens and the public authorities through a secure platform (see https://lifeindenmark.borger.dk/apps-and-digital-services/Digital-Post). Reminders are sent every 7 days. Foster children 10 years and older will receive the questionnaire via a text message. If the foster parent or the foster child needs help to fill out the questionnaire, they will receive a phone call or help from a member of the research team. Foster children will receive a DKK 150 (~ EUR 20) electronic gift card for each of the three data collections. The research team will closely monitor the data collection process. Data will be transferred to secure servers hosted by Statens IT. The data platform conforms to the international ISO27001 standard on how to manage information security. The trial statistician (MA), the principal investigator (MP), and the co-PI (NDT) will have access to the full dataset. We will not collect any biological data. Any adverse events will be monitored during the intervention and reported to the PI.

### Outcomes

Table 1 shows the timing of the administration of measures. Baseline data include foster parent background variables (e.g., age and education) and child variables (e.g., gestational drug/alcohol exposure, age at separation from biological parents, number of previous placements, length of the current placement, and significant mental/physical health problems). T1 is the baseline, T2 is 20 weeks post T1 baseline, and T3 is 6 months post T2.

**Table 1:** Outcomes

	Measure	Child age	T1	T2	T3
*Foster Parent measures*					
Background variables for foster parent			√		
Well-being	WHO-5 index		**√**	√	√
Parental Stress	PSS		**√**	√	√
Parental Reflective Functioning	PRFQ		**√**	√	√
*Child measures*					
Background variables for foster children		4–17	**√**		
Child psychosocial adjustment (caregiver)	CBCL	4–17	**√**	√	√
Child psychosocial adjustment (self-report)	SDQ self-report	10–17	**√**	√	**√**
Child Well-being	KIDSCREEN 10-index	8–17	**√**	√	**√**
Child attachment	SSAP	4–9	**√**	√	
*Relationship measures*					
Parental Mind-mindedness	Describe your child procedure		**√**	**√**	
Child Mind-mindedness	Describe your friend procedure	10–17	**√**	**√**	
Coding interactive behavior (video)	CIB		**√**	**√**	
*Other outcomes*					
Placement breakdown	Administrative data			**√**	√

### Primary outcome

The Child Behavior Checklist (CBCL) measures psychosocial adjustment and is a component of the Achenbach System of Empirically Based Assessment (ASEBA) [51, 52]. The ASEBA is used to detect behavioral and emotional problems in children and adolescents. The CBCL consists of 113 questions, scored on a three-point Likert scale (0 = absent, 1 = occurs sometimes, 2 = occurs often). Foster parents for all children in the trial will complete the CBCL. The CBCL/6–18 (used with children 6 to 18 years old) consists of eight syndrome scales: (1) anxious/depressed, (2) depressed, (3) somatic complaints, (4) social problems, (5) thought problems, (6) attention problems, (7) rule-breaking behavior, and (8) aggressive behavior. The syndrome scales group into two higher-order factors: (1) internalizing and (2) externalizing. The timeframe for item responses is the past six months. The CBCL also includes competence scales for activities, social relations, school, and total competence. The CBCL is widely used and has proven to be a useful tool for detecting psychopathology in children and shows good results regarding both validity and reliability [53]. Danish norms are available for children throughout the age range of children within the trial [51, 52]. A low score on the Total Problem Scale is beneficial.

### Secondary outcomes

Kidscreen-10 is a ten-item parental and self-report measure of child well-being (health-related quality of health) [54]. Items are scored from 1 (never) to 5 (always) except for items 1 and 9 (reverse). Items 1 and 2 explore the level of the child’s/adolescent’s physical activity, energy, and fitness. Items 3 and 4 cover how much the child/adolescent experiences depressive moods and emotions, and stressful feelings. Items 5 and 6 ask about the child’s/adolescent’s opportunities to structure and enjoy their social and leisure time and participation in social activities. Item 7 explores the quality of the interaction between the child/adolescent and parent or caregiver and the child’s/adolescent’s feelings toward their parents/caregivers. Item 8 examines the nature of the child’s/adolescent’s relationships with other children/adolescents. Finally, items 9 and 10 explore the child’s/adolescent’s perception of their cognitive capacity and satisfaction with school performance. A higher score is better [54]. Kidscreen-10 is used as a self-report measure for children aged 10 or above, while the parental rating version is used within all families.

The Strengths and Difficulties Questionnaire (SDQ) [55, 56] for 11–17-year-old children is a 25-item measure of child behavior and psychopathology. Items are rated by the foster child on a three-point scale (not true, somewhat true, certainly true). The SDQ consists of five domains: hyperactivity/inattention, peer problems, conduct problems, emotional symptoms, and pro-social behaviors. The SDQ also has an additional seven-item impact supplement about daily function. Foster children from 10–17 years old will receive the SDQ. A high score indicates more problems.

The Parental Reflective Functioning Questionnaire (PRFQ) is an 18-item measure of parental reflective function or mentalization [57]. The PRFQ consists of three subscales. The first subscale: Pre-Mentalizing Modes (PRFQ-PM) contains 6 items (a low score indicates better function). The second subscale: Certainty about Mental States (PRFQ-CMS) contains 6 items (a high score indicate better function), and the third subscale: Interest and curiosity in mental states (PRFQ-IC) also contains 6 items (a high score indicate better function) [57]).

The Parental Stress Scale (PSS) [58, 59] is an 18-item measure of parental stress that is rated on a five-point scale (Strongly disagree, Disagree, Undecided, Agree, Strongly agree). Total score range is from 18 to 90, where a low score indicates less stress.

The WHO-5 index [60, 61] is a five-item questionnaire assessing the emotional well-being of foster parents. The WHO-5 index can also be used as a screening tool for symptoms of depression. It consists of five positively formulated items. The degree to which these feelings were present in the two weeks prior to completing each questionnaire will be scored on a 6-point Likert-type scale ranging from 0 (not present) to 5 (constantly present). Item scores are summated and transformed to a 0–100 scale, with lower scores indicating poorer well-being. The WHO-5 index has been cross-culturally validated and has proven to be psychometrically sound [60, 61].

Family functioning will be measured by six items inspired by the General Functioning subscale of the Family Assessment Device (FAD-GF). The FAD-GF assesses overall healthy functioning or dysfunction of intra-familial relationships. The six items were selected to reflect the domains included in the McMaster Model of Family Functioning: problem-solving, communication, roles, affective responsiveness, affective involvement, and behavioral control [62]. In order to make items comprehensible to younger children, some items were slightly rephrased. Higher scores indicate greater family dysfunction.

#### Parent–child relationship

Coding Interactive Behavior (CIB) [63] is a global video-based coding system that assesses the foster parent–child relationship. The CIB includes 44 rating scales coded from 1 (a little) to 5 (a lot); 21 are parent codes, 16 are child codes, five are dyadic codes, and two are overall codes, which aggregate into the following higher-order composites: maternal sensitivity, maternal intrusiveness, child social engagement, and dyadic reciprocity. The system has versions for newborns, infants and toddlers, preschoolers, and adolescents. The CIB is coded based on a six-minute video recording of free play or interaction between parent and child in the home. The CIB system has been validated as an assessment measure in multiple studies of mother–child interactions in both normative and high-risk populations. It shows stability over time, predictive validity, and adequate psychometric properties [63]. In the present trial, the video coding of CIB will be based on a video of the child and the primary foster parent. Interactions will be coded by reliable coders blind to treatment allocation. The inter-coder agreement will be calculated on a 10% randomly selected subset of the sample.

#### Child attachment

The Story Stem Assessment Profile (SSAP) is a narrative-based measure to assess internal representations in 4-to-11-year-old children [25, 64]. Using a standard doll family and play materials, the interviewer enacts the beginning of a story (a story stem) and asks the child to complete the story using the provided play materials. The method allows the child to enact the story in a playful manner creating a narrative based on both verbal and non-verbal inputs, offering a unique insight into the child’s perception of the nature and quality of relationships. The SSAP entails a set of 13 story stems, which introduce the beginning of a story for the child to complete, within which lies "an inherent dilemma". It "allows an assessment of the child's expectations and perceptions of family roles without asking the child direct questions about their own family, which might cause the child undue conflict or anxiety. A shortened version of the SSAP consisting of seven stories, which has demonstrated robust psychometric properties and has been validated with looked-after children in the UK, will be used in the trial [25, 64]. Attachment will be coded by reliable coders blind to treatment allocation. The inter-coder agreement will be calculated on a 10% randomly selected subset of the sample.

#### Parental mind-mindedness

Parental mind-mindedness refers to a caregiver’s attunement to their children’s mental and emotional states [65]. In infancy, mind-mindedness is assessed from caregivers’ tendency to comment appropriately on their infants’ thoughts or feelings or from caregivers’ meaningful interpretations of their infants’ early non-word vocalizations [66]. In children beyond infancy, mind-mindedness is assessed in terms of parents’ tendency to spontaneously focus on mental characteristics when given an open-ended invitation to describe their child. In the present study, foster parents will be asked to complete the describe-your-child measure online [67], and coding will be carried out as described in previous studies with children in foster care [66]. Mind-mindedness will be coded by reliable coders blind to treatment allocation. The inter-coder agreement will be calculated on a 10% randomly selected subset of the sample.

#### Child mind-mindedness

Child mind-mindedness refers to children's tendency to adopt an intentional stance in their interactions with and representations of others, measured as their proclivity to use mentalistic descriptors in their representation of others [68, 69]. Mentalistic descriptors refer to any references to mental life and intellect (e.g., 'she is always thinking about him', 'he is a clever person'). References to likes and dislikes are only included if they do not focus exclusively on the friend's behavior (e.g., 'she likes blue' and 'she doesn't like her brother or her sister using her stuff’ are coded as mentalistic, but ‘she likes playing football’ is coded as behavioral). Also included in mentalistic descriptors are references to the friend's responses to the child's emotions (e.g., 'she plays with me when I'm feeling sad'), and to the friend's own emotions, but not their external manifestations (e.g., 'she's always really happy', but not 'always smiling'. In the present study, foster children aged 10 or above will be asked to complete the describe-your-friend measure online, and coding will be carried out as described in the manual [68, 69]. Mind-mindedness will be coded by reliable coders blind to treatment allocation. The inter-coder agreement will be calculated on a 10% randomly selected subset of the sample.

### Blinding

As the participants will be offered extra support in the intervention group, neither participants nor foster care consultants can be blinded. Data analysts, video coders, and data assessors will be blind to allocation status.

### Power considerations

Based on previous research, we expect an effect size of *d* = 0.53 for children’s overall psychosocial adjustment [40]. Based on 23 foster care consultants in each group, a power of 0.80, a type 1 error rate of 0.05, and an intra-class correlation (ICC) of 0.1, we need to include 3 families for each foster care consultant in the study (a total of 138 families). We aim to recruit 175 families (3.8 for each foster care consultant) to leave room for drop-out and allow power to conduct moderator analyses.

### Statistical analysis

We will estimate the mean treatment effect of MBT relative to UC on the primary outcome (CBCL) as well as each of the secondary outcomes (Parental Well-Being, Stress, and Reflective Functioning, SDQ, Kidscreen, SSAP, and Parental Mind-Mindedness) at T2 and T3 separately, but using the same method each time, as follows.

Treatment assignment will be stratified into municipalities, and within each stratum, it will be assigned to clusters based on foster care consultants. Hence the estimation needs to account for both the stratification and the clustering. We will thus use linear regression for T2 and T3 outcomes separately, in each case controlling for pre-treatment outcomes. Within each stratum, we will then use linear regression, estimating robust clustering standard errors in order to account for the potential correlation between subjects assigned to the same consultant. Using the strata-specific treatment effects, we will then compute the mean treatment effect as the weighted mean of the within-stratum estimates weighted by stratum share. We can calculate the variance by averaging the within-stratum cluster robust variance, weighted by stratum share squared [70]. All analyses will use a significance level of 0.05.

Analyses will be based on the intention-to-treat (ITT) principle, analyzing each subject as part of the treatment to which they were originally assigned, regardless of whether treatment was in fact received.

If there is substantial attrition in outcome data collection, this will potentially introduce selection bias in the estimated effect if attrition selection is on unobservables. While doing so does not solve the problem of attrition selection on unobservables, we will follow the standard in the literature and analyze sensitivity by handling missing values using multiple imputation using all baseline variables.

Further, we will perform subgroup analysis in order to examine whether treatment effects differ across subgroups based on age (4–9 and10–17 years), number of previous foster care placements, municipality budget per capita (low or high) as well as parental background variables.

### Interviews and observations

A central component of the MBT model is to enhance reflective capacities in the treatment environment around the foster child to ensure a professional, reflective relationship between foster families and foster care consultants. Therefore, we will apply qualitative methods to examine how reflective processes are implemented in practice. Through interviews and observations, we will examine how the implementation of MBT promotes and facilitates reflective capacities and practices in foster care consultants and in their interactions with foster parents. We will apply a case study design to help us clarify the influence of the context [71]. Data will be collected in four municipalities that differ in critical strategic characteristics (e.g., size, rural/urban). Across the four municipalities, we will look for similarities and differences in the implementation of MBT. Finding regularities across different municipalities will be seen as an indicator of robustness [71].

Data will consist of: (1) video recordings for use in foster care consultant’s supervision, (2) observational data from sessions with foster families and from supervision sessions with MBT supervisors, and (3) semi-structured interviews with foster care consultants. In each municipality, we will collect data from four foster care consultants (two MBT therapists and two control therapists), providing data from 16 foster care consultants in four local contexts.

We will collect video recordings from virtual group supervision sessions (one per group) for MBT therapists. We will conduct two observations and interviews with each MBT and control therapist. Observations and interviews will be conducted in the beginning of the intervention when the focus of intervention is being established and again after four to eight weeks (by the end of the intervention). The observations will be conducted as non-participatory observations based on a semi-structured observational framework [72]. The interviews will be individual and conducted in person.

All interviews and observations will be audio-recorded and transcribed verbatim. Data will be analyzed based on thematic analysis [73] including the following steps: getting familiar with the data and generating initial codes, followed by a process where we search, review and name themes [73]. We expect that themes will include reflective reasoning, changes in the work procedures, and cooperation with foster families. NVivo will be used as analytical software in this process.

## Discussion

This prospective cluster-randomized controlled trial aims to explore the effects of MBT for foster families and foster children aged 4–17 years old in Denmark on measures of child mental health and well-being, foster/parent–child relationship and attachment, as well as foster parent stress and mental health. Furthermore, follow-up measures included in the trial may enable analyses of the effectiveness of the MBT intervention in preventing placement breakdown, which is a known risk factor for long-term positive outcomes for children in foster care. To our knowledge, this is the first randomized controlled study examining the effects of an attachment-based intervention on foster families in the Scandinavian context. This is paradoxical, as the importance of attachment theory within foster care is often seen as taken-for-granted knowledge in official documents and clinical guidelines [17].

In Denmark, there are rather large differences between the municipalities in terms of sociodemography and population size, which results in large differences regarding the number of children placed in care within each municipality. This means that municipalities with relatively many children in care also have a high number of highly skilled foster care consultants, or they may already have psychologists or family therapists who provide therapeutic interventions for foster families in times of high stress. On the other hand, some municipalities with more budgetary restrictions or with very few children in care may only employ a few foster care consultants. Consequently, the practical execution of the study will be streamlined as much as possible but with a high degree of respect for what is actually possible within the participating.

The MBT therapists who receive the training will vary in terms of educational background and clinical experience. This is likely to cause variation in the delivery and receipt of the MBT intervention. The study will provide insight into these differences, as we will be able to conduct moderator analysis based on therapist characteristics.

Other potential limitations relate to methodological as well as practical challenges. A practical challenge concerns the probability of attrition among foster families allocated to the control group. If MBT is perceived as an attractive offer, being assigned to UC while still spending time answering questionnaires and providing a video of foster parent/child interaction to be used for research might be difficult to accept. Because we cannot apply strict random allocation, comparability between the intervention group and control group cannot be guaranteed, and conclusions about causality are expected to be tentative rather than definitive. On the other hand, the study is conducted in a naturalistic setting, which can be regarded as a strength as it increases the ecological validity of our findings and the likelihood that the municipalities may decide to continue to offer the MBT intervention after the end of the trial.

## Data Availability

To protect participant privacy, the datasets generated and analyzed during the current study are not publicly available, but are available from the corresponding author upon reasonable request.
